# A Just Digital framework to ensure equitable achievement of the Sustainable Development Goals

**DOI:** 10.1038/s41467-021-26217-8

**Published:** 2021-11-03

**Authors:** Katriona O’Sullivan, Serena Clark, Kevin Marshall, Malcolm MacLachlan

**Affiliations:** 1 Assisting Living and Learning Institute, Dept. of Psychology, Maynooth University, Kildare, Ireland; 2 Microsoft Education Ireland, Microsoft Place, Ireland

**Keywords:** Technology, Developing world, Careers, Education, Ethics

## Abstract

While the technological revolution is accelerating, digital poverty is undermining the Sustainable Development Goals. This article introduces a justice-oriented digital framework which considers how fair access to digital capabilities, commodities, infrastructure, and governance can reduce global inequality and advance the SDGs.

The United Nations Sustainable Development Goals (SDGs) are an ambitious global initiative identifying grand challenges partitioned into 17 goals, each with specific sub-targets. The key to these goals making a real difference to people’s lives is that they are pursued in an integrated way, that this pursuit should “Leave No One Behind”. Global support for these goals was a heart-warming assertion of our valuing of humanity collectively and our shared commitment to acting together. However, technological advancements in the last years have introduced a significant threat to their collective achievement, a point reiterated by the UN Secretary-General in 2020; “Of the SDG’s 17 goals and 169 targets, not a single one is detached from the implications and potential of digital technology…”^1^

The digital divide is egregious to the ethos and the reality of achieving the SDGs. More than four billion people lack access to the internet in emerging economies^2^. There are shocking disparities between those who have and those who have no access to the wealth that digital access brings. In India, 50% of the population has access to the internet even though it is a country with the second-largest online market. And 66% of Caucasians have access to high-speed internet, compared to 49% of African Americans^2^. Control over digital access is power and can be dangerous. Recent times have seen internet shutdowns by governments or power outages in Ethiopia, Zimbabwe, Azerbaijan, Iraq, Iran, and Myanmar for political or economic reasons^3^. Such shutdowns reduce access to life-saving information, restricting freedom of expression and association. They make it hard to organize opposition and cost economies billions. Now is the time for society to reflect on how these forms of digital poverty impact achieving the SDGs, identifying how the Fourth Industrial Revolution’s technological advancements can ensure that no one is left behind.

## Digital divide and SDG achievement

Developing the digital capabilities necessary to utilize technology meaningfully is essential for achieving the SDGs. SDG 1 aims to ensure that all men and women, particularly the poor and the vulnerable, have equal rights to economic resources that reduce poverty. In recent times we have seen that having the capacity to navigate the digital world has been central to maintaining employment, education, and connection^4^. Through COVID19, even the wealthiest countries have fallen short in ensuring that their citizens had the appropriate technologies and were skilled enough to maintain meaningful engagement in their everyday lives.

Just as fast as digital access and new and remarkable digital technologies have accelerated learning and knowledge for many, the lack of access, commodities, and capabilities leaves behind many more^4^. This is not only about access to technology but more fundamentally about people lacking the capabilities to use technology to engage in modern living. The emerging skills gap is evidence of this^5^ and the lack of integrated digital systems is burdening economies, widening the space between the rich and the poor, and preventing society from achieving SDG 1. Amartya Sen’s Human Capabilities Approach^6^ states that the human welfare derived from digital commodities depends not only on ownership and access but also on what can be done with such goods. In the context of ensuring we achieve all SDGs, having access to education (SDG 4), work (SDG 9), health (SDG 3), wealth (SDG 1), and security (SDG 16), requires society to provide *both* digital commodities and the digital capabilities needed to garner the benefit of the technological revolution.

SDG 9 focuses on building resilient infrastructures, promoting inclusive and sustainable industrialization, and fostering innovation. However, we are not equally empowered to engage in modern society in a way that supports innovation and growth. In less than 20 years, commercial internet has become a requirement for full participation in society, and the digital economy has become a digital ecosystem in itself. This ecosystem comprises multiple stakeholders, ranging from corporations to governments to NGOs and international organizations like the United Nations. As there is an increased dependence on the internet, companies create new services to meet these needs, like the Internet of Things, the Cloud, and machine-to-machine learning^7^, making accessible digital infrastructure critical for the achievement of SDG 9.

When considering who has meaningful access to emerging technologies and developing infrastructures, we begin to see a new face of inequality emerging. For example, technologies like AI and blockchain offer vast opportunities to reduce inequalities. One of the commonly known applications of blockchain is crypto-currency which has been successfully used as an alternative financial sector in emerging economies^8^. People experiencing health disparities have also benefited from it in terms of gaining safe access to health records, telehealth and reducing the spread of misinformation about medical treatments and vaccines^9^. However, technical know-how is necessary to garner the full benefit from blockchain.

In low or middle-income countries, there is limited access to digital infrastructures, which can reduce inequalities. Being absent from data sets that underpin developing technologies increases the risk of marginalization. This has been seen in AI programs that determine sentences for criminals, which are biased against African Americans^10^ or motion vision algorithms that do not work as effectively on darker-skinned people as lighter-skinned people^11^. In both cases, AI algorithms were developed using data sets with an overrepresentation of light-skinned people. As it currently stands, there is no SDG dedicated explicitly to digital systems; only four explicitly reference the impact that emerging Information Communication Technology (SDGs 4, 5, 9, 17) can have on inequality and the broader achievement of the SDGs^12^. The European Commission has highlighted this point, warning that while growth in AI, blockchain and digitalization has the potential to advance society, it also risks perpetuating historical inequalities in society, particularly where there are data gaps along the lines of class, gender, rac, or ethnic lines^13^.

Access to digital technologies also facilitates financial inclusion, mobile banking, microcredits, remittances, and increases market access^14^. These significantly contribute to alleviating poverty by highlighting the needs of vulnerable groups using real-time analytics and data. Simultaneously, digital systems allow vulnerable populations to partner with and co-create solutions with a wide range of stakeholders. Digital finance, e-commerce, and ethical e-governance innovations are increasing access to information and services. For example, China is seeing a decrease in the rural-urban divide due to using e-governance to increase access to health, education, and non-agricultural income-generating opportunities in rural areas^12^. In Bangladesh, iFarmer, a digital crowdfunding platform, gives rural women cattle farmers more access to markets by allowing investors to offer them capital^12^. Meanwhile, Rohingya refugees are creating livelihoods in Cox Bazaar using the e-commerce platform ekShop Shoron^12^.

While digital skills are named in SDG 4, as a target that seeks to increase the number of people with relevant skills for employment^7^. These indicators only focus on relevant skills, ignoring the commodities and infrastructures which impact their development and use. And reflection upon their growth is based solely on self-reported information of countries, with analysis showing perpetuation of inequalities through skewed data sets and reporting biases across gender groups, with women under and men overreporting^15^.

## The Just Digital framework

To address the rapidly changing world and the increasing role of the digital realm in everyday lives, we propose the Just Digital ethical framework to help progress the SDG 2030 Agenda. Just Digital has identified four target areas with associated indicators, which can be used to sense check how they affect the achievement of the SDGs and how they can facilitate future success. The target areas are implicated in all SDGs to varying extents; they include equity, education, employment, and engagement. The framework proposes that there are four interrelated digital concepts that, if prioritized and supported across all of society, will ensure success in the targeted areas, and equitable achievement of the SDGs. The digital four concepts include;

1. *Digital Infrastructure*—a working communications system that allows everyone to get online and access work, school, and life remotely and equally. This positively impacts economic and social outcomes through access to modern work and education and representation in data algorithms in development.
2. *Digital Capabilities*—an education system that provides everyone with the capacity to navigate the digital world and to engage in education, the economy, and the social world. This should include all of society, especially those who are on the margins, and it should be an expectation for life-long learning—of continuing personal development.
3. *Digital Commodities*—providing access to the hardware needed to engage in a basic standard of living.
4. *Digital Governance*—an ecosystem that supports the integration of digital technologies into daily living, ensuring that there are national policies which are integrated, which protect citizens’ rights, which upholds confidentiality, and which are imbued with ethical safeguards and security, not just regarding individuals but also our responsibility to others, through digital social inclusion. This governance should also consider the ethics of data infrastructure and ownership.

Table 1 provides an outline of how the Just Digital framework weaves across existing targets for other SDGs and supports the achievement of the four target areas, which are at the core of the 17 SDGs. Target area one, equity, is implicated in several SDGs, including SDG 9 that targets innovation and SDG 10, which focuses on reducing inequalities. The first cell in Table 1 highlights the ways in which working digital infrastructures can reduce inequities across several of the SDGs. This translates into supporting the achievement of SDG 10, reducing inequalities, and SDG 9, increased innovation. Using examples related to SDG 10, we see that in developing countries, the lack of good digital infrastructure results in minority groups being absent from data sets, which underpin developing technologies. This perpetuates inequalities through the production of biased AI systems. By setting specific targets to ensure digital infrastructures are in place across all of society, we decrease the risk of inequities; by increasing access to digital infrastructures, we can ensure that light- AND dark-skinned people are included in emerging AI^10^, which support the achievement of SDG 10. This is one of many ways in which the Just Digital framework can be utilized to reflect upon how digital advances can progress the SDGs core targets.

**Table 1:** Reflection of how the Just Digital framework, in four key impact areas, can facilitate achievement of targets and related SDG’s.

Just Digital framework				
	Equity	Education	Employment	Engagement
Digital infrastructure	Allows accurate representation of data in emerging technologies, algorithms, and AI and ensures equal access to employment and education opportunities	Provides infrastructure for learners to engage in modern economy and develop skills for modern living	Provides capacity to connect remotely, to work in preferred locations, and to collaborate across communities and countries.	Allows for access to health, wellbeing and connection with community particularly for those with disabilities or other forms of marginalization.
SDGs	1,3,4,5,8,9,10,16,17	1,3,4,5,8,9,10,16,17	1,3,4,5,8,9,10,17	3,5,10,16,17
Digital capabilities	Provides equal access to twenty-first-century skills including those living in poverty, females, aged, and those underrepresented in modern economy.	Empowers learners to emerge from education with skills needed to engage in modern society.	Empowers society to engage in workforce, reducing poverty and increasing creativity and connection.	Allows digital health, wellbeing, and connection tools to be utilized to their full potential
SDGs	1,3,4,7,8,10,17	1,4,5,7,8,10,17	1,4,5,7,8,10,17	1,4,5,7,8,9,10,17
Digital commodities	Provides all of society with technology and hardware to engage in modern society. Provides technology for accurate representation of data in developing technologies.	Provides education with the technological capacity to ensure learners are digitally prepared and digitally empowered	Provides access to technologies and hardware for remote working, and for connection and collaboration. Empowers workers to use technologies to enhance their work-life experience	Provides societal access to technologies and hardware, which support health, wellbeing, and connection for all
SDGs	1,3,4,7,8,10,17	1,3,4,7,8,9,17	3,4,8,10,16,17	1,4,5,8,9,10,11,17
Digital Governance	Policies to ensure equal access to digital resources, digital society use of data, representation, and equality in digital data development	Develops international framework of digital skills which empowers learners to engage in modern society and is recognized across the world for ease of movement	Develops collaborative international policies with industry which focus on equality, fairness, data ownership, data sharing, and digital society for good	National policies which protect citizens rights, which upholds confidentiality, and which are imbued with ethical considerations.
SDGs	3,4,8,10,16,17	1,4,5,8,9,10,16,17	3,5,7,8,10,16,17	3,10,16,17

Table key-description of the Sustainable Development Goals.

SDG 1 No poverty → SDG 2 Zero hunger.

SDG3 Good health and wellbeing → SDG 4 Quality education.

SDG 5 Gender equality → SDG 6 Clean water and sanitation.

SDG 7 Affordable and clean energy → SDG 8 Decent work and economic growth.

SDG 9 Industry, innovation and infrastructure → SDG 10 Reduced inequalities.

SDG 11 Sustainable cities & communities → SDG12 Responsible consumption.

SDG 13 Climate action → SDG 14 Life below water.

SDG 15 Life on land → SDG 16 Peace.

SDG 17 Partnerships for the goals.

The Just Digital framework has the potential to buffer against the socially corrosive effects of inequitable achievement of the SDGs, which is currently leaving behind and oppressing the disconnected. The framework provides a new way to consider the achievement of the SDGs. It provides a lens to consider how the digital revolution can ensure the ethical achievement of these goals. Table 1 has taken four target areas identified through analysis of the SDGs, and established key areas where the four interrelated digital concepts can ensure that the SDG 2030 Agenda is on track, and that it is taking into consideration the digital revolution. Just Digital is a starting point; it is a justice-orientated, ethical framework, which can help society to reflect on how and why digital is being used, and what the likely consequences of its use are for society. Such reflection will ensure digital poverty is reduced, and help to ensure that the SDGs are harnessing the value of the technological revolution^16,17^.

## Supplementary information

Editor Summary

## References

[1] Microsoft. 2020. Microsoft and the United Nations Sustainable Development Goals. *Microsoft*. https://query.prod.cms.rt.microsoft.com/cms/api/am/binary/RE4GSkV (2020).

[2] Shenglin, B., Simonellie, F., Ruidong, Z., Romain, B., and Li, W. Digital infrastructure: overcoming digital divide in emerging economies. Policy Brief, G20 Insights. https://www.g20-insights.org/wp-content/uploads/2017/05/Digital_Overcoming-Digital-Divide-II.pdf (2017).

[3] Duggal, H. *Mapping Internet Shutdowns around the World* (accessed 30 April 2021); https://www.aljazeera.com/news/2021/3/3/mapping-internet-shutdowns-around-the-world (2021).

[4] O’Sullivan K (2020). “A qualitative study of child and adolescent mental health during the COVID-19 pandemic in Ireland.”. Int. J. Environ. Res. Public Health.

[5] Zucchetti, A., Cobo, C., Kass-Hanna, J., and Lyons, A. C. *Bridging the Gap Between Digital Skills and Employability for Vulnerable Populations*. G20 Insights. https://www.g20-insights.org/policy_briefs/bridging-the-gap-between-digital-skills-and-employability-for-vulnerable-populations (2019).

[6] Hoque MR (2020). The impact of the ICT4D project on sustainable rural development using a capability approach: evidence from Bangladesh. Technol. Soc..

[7] Mondejar, M. E. et al. Digitalization to achieve sustainable development goals: Steps towards a Smart Green Planet. *Sci Total Environ.* **794**, 148539 (2021). 10.1016/j.scitotenv.2021.148539 34323742

[8] Vincent O, Evans O (2019). Can cryptocurrency, mobile phones, and internet herald sustainable financial sector development in emerging markets?. J. Transnatl Manag..

[9] Vervoort D, Guetter CR, Peters AW (2021). Blockchain, health disparities and global health. BMJ Innov..

[10] Knight, W. *Forget Killer Robots—Bias Is the Real AI Danger* (accessed 5 May 2021); https://www.technologyreview.com/2017/10/03/241956/forget-killer-robotsbias-is-the-real-ai-danger (2017).

[11] Lonescu, D. *Is Microsoft’s Kinect Racist?* (accessed 5 May 2021); https://www.pcworld.com/article/209708/Is_Microsoft_Kinect_Racist.html (2010).

[12] UNESCAP. *Inequality in Access to Information and Communication Technologies (ICTs) in East and North-East Asia and South-East Asia* (United Nations, New York, 2020). https://www.unescap.org/sites/default/d8files/knowledge-products/ESCAP_InequalityICT.pdf.

[13] European Parliment. *T**he Ethics of Artificial Intelligence: Issues and Initiatives*. (European Parliamentary Research Service, 2020); https://www.europarl.europa.eu/RegData/etudes/STUD/2020/634452/EPRS_STU(2020)634452_EN.pdf.

[14] ITU. *Digital Financial Services: Regulating for financial inclusion - an ICT Perspective*. (ITU, 2016); https://www.itu.int/dms_pub/itu-d/opb/pref/D-PREF-BB.REG_OUT02-2016-PDF-E.pdf.

[15] Montoya, S. *Meet the SDG 4 Data: Indicator 4.4.1 on Skills for a Digital World* (accessed 18 May 2021); https://sdg.uis.unesco.org/2018/08/08/meet-the-sdg-4-data-skills-for-a-digital-world (2021).

[16] MacLachlan, M. and McVeigh, J. eds. 2021. *Macropsychology: a Population Science for Sustainable Development Goals* (Springer, New York, 2021).

[17] United Nation. *With Almost Half of World’s Population Still Offline, Digital Divide Risks Becoming ‘New Face of Inequality’, Deputy Secretary-General Warns General Assembly* (accessed 30 April 2021); https://www.un.org/press/en/2021/dsgsm1579.doc.htm.

