# Supplementary material for: A Just Digital framework to ensure equitable achievement of the Sustainable Development Goals
Source: Nat Commun. 2021 Nov 3;12:6345. doi: 10.1038/s41467-021-26217-8 (PMC8566573; doi:10.1038/s41467-021-26217-8)
Supplement: Supplementary file 1 — Editor Summary [file 41467_2021_26217_MOESM1_ESM.docx]

Despite technological advances, the Sustainable Development Goals are undermined by global digital inequality. Enhancing digital capabilities, commodities, infrastructure and governance may help in reducing inequality and advancing the SDGs.
